# O11.8. PREVALENCE AND PREDICTORS OF INTERVIEW-ASSESSED CLINICAL HIGH-RISK SYMPTOMS IN THE GENERAL POPULATION

**DOI:** 10.1093/schbul/sby015.268

**Published:** 2018-04-01

**Authors:** Frauke Schultze-Lutter, Chantal Michel

**Affiliations:** 1 Heinrich-Heine University Düsseldorf; 2 University Hospital of Child and Adolescent Psychiatry and Psychotherapy, University of Bern

## Abstract

**Background:**

In clinical samples, symptomatic ultra-high risk criteria and the basic symptom criterion “cognitive disturbances” perform well in predicting psychosis, and best when both approaches are combined. However, little-to-nothing is known about the prevalence, clinical relevance, and moderators of these clinical high risk (CHR) criteria and their constituent symptoms in the community.

**Methods:**

Regression analyses involved 2683 community participants (age 16–40 years; response rate: 63.4%). Semi-structured telephone interviews were performed by well-trained psychologists.

**Results:**

Lifetime and current CHR symptoms were reported by 21.1% and 13.8% of interviewees. Frequency of symptoms was mostly low, only 2.4% met any CHR criterion. A stepwise relationship underlay the association of the two types of CHR symptoms and criteria with the presence of mental disorders and functional deficits, with odds ratios being highest (7.4–31.8) when ultra-high risk and basic symptoms occurred together. Report of a family history of mental disorder generally increased risk for CHR symptoms. While younger age increased risk for basic symptoms, lifetime substance misuse and trauma increased risk for ultra-high risk symptoms.

**Discussion:**

Prevalence of CHR criteria was within the range to be expected from the prevalence rates of psychoses. Clinical relevance of both CHR symptoms and criteria increased in a stepwise manner from basic symptoms via ultra-high risk symptoms to their combined presence, reinforcing the clinical utility of their combined use. The risk factors selectively associated with basic and ultra-high risk symptoms seem to support developmental models relating basic symptoms to neurobiological and ultra-high risk symptoms to psychological factors.

